# Using real-world data to inform dosing strategies of rituximab for pediatric patients with frequent-relapsing or steroid-dependent nephrotic syndrome: a prospective pharmacokinetic-pharmacodynamic study

**DOI:** 10.3389/fphar.2023.1319744

**Published:** 2024-01-09

**Authors:** Yewei Chen, Qian Shen, Ye Xiong, Min Dong, Hong Xu, Zhiping Li

**Affiliations:** ^1^ Department of Pharmacy, Children’s Hospital of Fudan University, National Children’s Medical Center, Shanghai, China; ^2^ Department of Nephrology, Children’s Hospital of Fudan University, National Children’s Medical Center, Shanghai, China; ^3^ Division of Clinical Pharmacology, Cincinnati Children’s Hospital Medical Center, Cincinnati, OH, United States; ^4^ Department of Pediatrics, University of Cincinnati, Cincinnati, OH, United States

**Keywords:** pharmacokinetics, pharmacodynamics, pediatric, nephrology, precision medicine

## Abstract

**Objectives:** Rituximab is frequently used off-label for the treatment of frequent-relapsing/steroid-dependent nephrotic syndrome (FRNS/SDNS). However, the optimal dosing schedules remain undetermined. The objective of this study was to establish a population pharmacokinetic-pharmacodynamic (PK-PD) model in pediatric patients with FRNS/SDNS, and to investigate dosing regimens that provide adequate suppression of B lymphocytes.

**Methods:** A prospective, open-label, single-center study was conducted in Nephrology Department at Children’s Hospital of Fudan University, and a two-compartment PK model of rituximab in pediatric FRNS/SDNS has been developed previously by our group. CD19^+^ lymphocyte count profiles were obtained from these patients. The presence of anti-rituximab antibodies was assessed prior to medication in children who had previously received rituximab or during follow-up at the last sampling point for PK analysis. PK-PD analyses were performed to describe the changes of CD19^+^ lymphocytes, with rituximab assumed to increase their death rate. Monte Carlo simulation was conducted to evaluate different dosing regimens.

**Results:** In total, 102 measurements of CD19^+^ lymphocyte counts were available for PK-PD analysis. No detectable levels of anti-rituximab antibodies were observed during the PK follow-up period. A turnover model with saturable stimulatory action of rituximab on the removal of lymphocytes best characterized the relationship between rituximab concentration and CD19^+^ lymphocyte counts, where the E_max_ and EC_50_ were estimated to be 99.6*10^6^/L and 5.87 μg/mL, respectively. Simulations indicated that a single infusion of 750 mg/m^2^ and 2 infusions of 375 mg/m^2^ both yielded a 10-week suppression of CD19^+^ lymphocytes.

**Conclusion:** This study represents a first attempt to quantitatively describe the PK-PD relationship of rituximab in pediatric patients with FRNS/SDNS, and provide a potential pathway for future precision dosing strategy for rituximab therapy. Further clinical studies are warranted to evaluate the efficacy and safety of different dosing schemes.

## Introduction

Rituximab, a chimeric monoclonal antibody initially approved for the treatment of B cell non-Hodgkin’s lymphoma (NHL) (FDA, 1997), has recently gained popularity for off-label use in managing frequent-relapsing/steroid-dependent nephrotic syndrome (FRNS/SDNS). Numerous studies have demonstrated the efficacy and safety of rituximab in maintaining remission and reducing or discontinuing the use of steroids and immunosuppressants in childhood FRNS/SDNS (Ravani et al., 2011; Iijima et al., 2014; Ravani et al., 2015; Ahn et al., 2018; Basu et al., 2018). Based on the findings in a pivotal clinical trial, Japanese government approved the use of rituximab in FRNS/SDNS patients (Iijima et al., 2014). The new Kidney Disease Improved Outcome (KDIGO) guidelines now recommend the use of rituximab for the treatment of FRNS/SDNS in pediatric patients (KDIGO Glomerular Diseases Work Group, 2021). After administration, rituximab rapidly binds to the CD20 antigen expressed on B cells, resulting in B cell depletion through various mechanisms such as complement-dependent cytotoxicity (CDC), antibody-dependent cell-mediated cytotoxicity (ADCC), antibody-dependent cellular phagocytosis, as well as direct effects via apoptosis or other cell death pathways (Weiner, 2010; Boross and Leusen, 2012; Maloney, 2012; Abulayha et al., 2014). The depletion of B cells induced by rituximab has potential to decrease the production of abnormal autoreactive antibodies that attack podocytes and result in proteinuria. Additionally, it may hinder the generation of co-stimulatory signals on activated T cells and cytokines that regulate T cell differentiation (Kallash et al., 2019; Sinha et al., 2021). B cell depletion has been used as a biomarker to assess the response to rituximab in nephrotic syndrome (NS), typically defined as less than 1% of lymphocytes. Numerous studies have demonstrated a strong correlation between B-cell depletion and sustained remission, with relapses commonly occurring following B cell recovery (Guigonis et al., 2008; Sel et al., 2012; Coluc et al., 2016). When assessing B cell counts, CD19^+^ cell counts in peripheral blood are typically monitored, as CD19 is expressed continuously from the early pre-mature B cells to the early stages of plasma cell differentiation.

However, the response to rituximab varies in patients with FRNS/SDNS (Kamei et al., 2009; Kemper et al., 2012; Ravani et al., 2013), and there is currently no standardized dosing regimen. In Japan, the use of 375 mg/m^2^ once weekly for 4 weeks has been approved for treating complicated FRNS/SDNS in both adult and pediatric patients (Iijima et al., 2014). In the United Kingdom, a national policy statement recommends administering two doses of 750 mg/m^2^ on day 1 and day 15 for patients weighing less than 50 kg (Specialised Commissioning Team, 2015). At our center, we routinely administer 375 mg/m^2^ once weekly for 2 weeks. The selection of an appropriate dosing regimen for pediatric patients with FRNS/SDNS remains a pertinent question. The initial dosing regimen of rituximab at 375 mg/m^2^ four weekly was borrowed from those in use for B-cell lymphoma (Benz et al., 2004; Gilbert et al., 2006). Subsequently, various dosing protocols have been reported, including single-dose, 2-dose, 4-dose and 5-dose protocols, with doses ranging from 100 to 750 mg/m^2^ per dose (Kamei et al., 2009; Kemper et al., 2012; Ahn et al., 2018; Hogan et al., 2019; Maxted et al., 2019). This wide range of variability has hindered the direct comparison of these different regimens across the reported studies, thereby preventing the establishment of the most appropriate dosing schedule for rituximab in FRNS/SDNS.

Population pharmacokinetic-pharmacodynamic (PK-PD) modeling and simulation have proven to be a useful approach to gain a better understanding of the dose-exposure-response relationship, to predict study outcomes prior to their implementation. Previously, our group developed a two-compartment pharmacokinetic (PK) model of rituximab in pediatric patients with FRNS/SDNS (Chen et al., 2021). It is worth noting that patients with FRNS/SDNS displayed distinct PK profiles compared to those with other indications. Specifically, patients with FRNS/SDNS exhibited a clinically significant increase in rituximab clearance when compared to the patient population with NHL. However, relying solely on the PK model alone cannot provide a comprehensive model-guided dosing strategy for therapy. The aim of this study was to develop a population PK-PD model of the effect of rituximab on eliminating B lymphocytes in the treatment of pediatric patients with FRNS/SDNS, and to investigate dosing regimens that provide adequate suppression of B lymphocytes (below 10 × 10^6^/L) while minimizing the dose. Furthermore, we assessed the presence of anti-rituximab antibodies prior to medication (in children who had previously received rituximab) or during follow-up (at the last sampling point for PK analysis).

## Materials and methods

### Data source

A two-stage prospective, open-label study of rituximab was conducted in the Nephrology Department at Children’s Hospital of Fudan University between January and July 2017. At stage 1, fourteen pediatric patients diagnosed with FRNS/SDNS were enrolled, and a population PK analysis has been previously described (Chen et al., 2021). At stage 2, peripheral blood samples were collected at each visit for all these patients and CD19^+^ lymphocyte counts in peripheral blood were measured. Additionally, anti-rituximab antibody concentrations were determined in the samples utilized for PK analysis. One out of the 14 children had received prior treatment with rituximab; therefore, pre-administration testing for anti-rituximab antibodies was conducted. All children underwent measurement at the last PK sampling point. The study protocol was approved by the Ethics Committee of Children’s Hospital of Fudan University, and written informed consent was obtained from each child’s parents or legal guardians prior to enrollment.

### Laboratory analysis

The concentrations of rituximab were determined using the SHIKARI^®^ enzyme-linked immunosorbent assay (ELISA) kit provided by Matriks Biotechnology Company Limited, as previously described (Chen et al., 2021). CD19^+^ lymphocyte counts in peripheral blood were measured by flow cytometry. Qualitative determination of anti-rituximab antibodies was performed using the SHIKARI^®^ S-ATR ELISA kit from Matriks Biotechnology Company Limited. Assay buffer was added to each well, followed by pipetting 10 µL of samples, reactive control, and negative control into their respective wells on microtiter plates. The plates were incubated at room temperature for 1 h and then washed three times. Ready-to-use peroxidase conjugate (100 µL) was added to each well and incubated at room temperature for 1 h. After incubation, the plates were washed three times as before. Tetramethylbenzidine substrate solution (100 µL) was added to each well and incubated at room temperature in the dark for 20 min. The substrate reaction was stopped by adding stop solution (100 µL) to each well, resulting in a color change from blue to yellow indicating completion of the reaction. Optical density measurement was taken at 450 nm within 30 min after addition of stop solution using a multi-plate reader (Synergy 2, BioTek, Winooski, VT, United States). The positive control should exhibit an OD450 nm value of ≥1.00 while each negative control should have an OD450 nm value of <0.150. A sample is considered positive if the ratio of sample OD_450_ to the mean OD_450_ of negative controls is ≥ 3; otherwise it is classified as negative.

### Population pharmacokinetic-pharmacodynamic modelling

**FIGURE 1:**
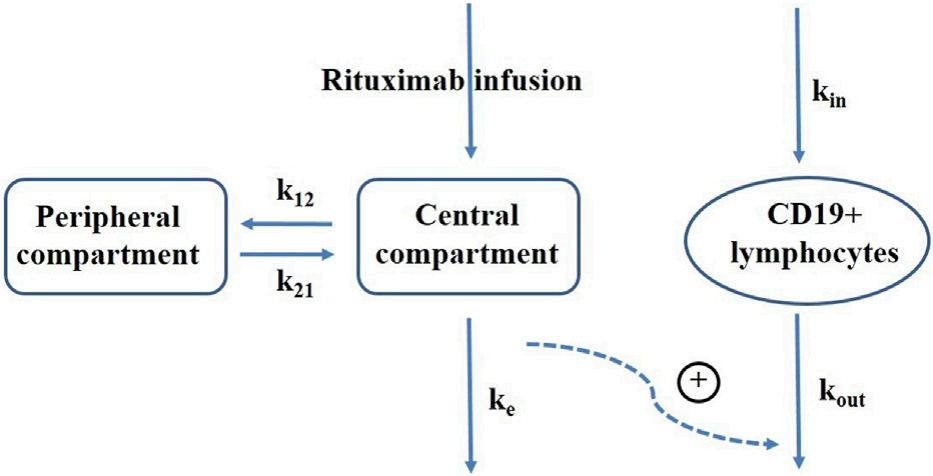
Schematic representation of rituximab pharmacokinetic-pharmacodynamic model. k_12_, elimination rate constant from the central compartment to peripheral compartment; k_21_, elimination rate constant from the peripheral compartment to central compartment; k_e_, elimination rate constant of rituximab; k_in_, production rate of CD19^+^ lymphocytes; k_out_, elimination rate constant of CD19^+^ lymphocytes; plus sign, promotion on CD19^+^ lymphocytes elimination.

A sequential population PK-PD model was developed using NONMEM program (version VII, Icon Development Solutions, Ellicott City, MD, United States of America) to describe the temporal changes in CD19^+^ lymphocyte counts following rituximab treatment. The PK model employed a two-compartment structure with body surface area as a significant covariate (Chen et al., 2021). A turnover model was constructed to capture the dynamics of CD19^+^ lymphocytes considering the impact of rituximab on increasing their death rate. Figure 1 presents a schematic representation of the population PK-PD model. Equations for the PK-PD model are provided as follows (Eqs 1–4):

$$C1=A\left(1\right)/V1$$ (1)

$$dA1/dt=k21\times A\left(2\right)-k12\times A\left(1\right)-CL\times C1$$ (2)

$$dA2/dt=-k21\times A\left(2\right)+k12\times A\left(1\right)$$ (3)

$$dA3/dt=Kin-Kout\times \left(1+EMAX\times C1/\left(EC50+C1\right)\right)\times A\left(3\right)$$ (4)

Where C1 represents the concentration in the central compartment, which is initially zero prior to dosing; A_1_ and A_2_ represent the amounts of rituximab in the central and peripheral compartments respectively, also starting at zero when t = 0; V_1_ denotes the central volume of distribution; k_12_ represents the elimination rate constant from the central compartment to peripheral compartment; k_21_ represents the elimination rate constant from the peripheral compartment to central compartment; CL represents the central clearance; A_3_ represents CD19^+^ lymphocytes; k_in_ represents the production rate of CD19^+^ lymphocytes; k_out_ represents the elimination rate constant of CD19^+^ lymphocytes; E_max_ corresponds to maximum increase in cell elimination rate with rituximab presence; EC_50_ refers to concentration producing 50% of maximum effect. In absence of rituximab, baseline CD19^+^ count is given by k_in_/k_out_, from which we derived k_in_ by estimating baseline and k_out_ as parameters. The baseline parameter was used for initializing CD19^+^ lymphocytes compartment. Inter-individual variability was assumed to follow a log-normal distribution and described using exponential model. Proportional, additive and combined proportional and additive residual error models were tested for describing unexplained residual variability.

Covariates investigated for their influence on PD model parameters included baseline characteristics (gender, age, weight, height, body surface area), laboratory values (albumin, total cholesterol, serum creatinine, creatinine clearance, cystatin C and blood urea nitrogen) and disease state (age at onset, NS duration, proteinuria, proteinuria-to-creatinine ratio) (Chen et al., 2021). Continuous covariates were described using the exponential function while categorical covariates were modeled using a power function. The stepwise covariate modeling method with forward selection (*p* = 0.05) and backward elimination (*p* = 0.01) based on the likelihood ratio test was employed to identify statistically significant covariates.

### Model evaluation

The model fit was assessed graphically using goodness-of-fit plots, including observed CD19^+^ lymphocyte counts *versus* predictions for the population or individual patients, as well as conditional weighted residuals *versus* time or population predictions. Bootstrap analysis was performed for the final model with 1,000 randomly resampled data sets from the original dataset (Ette et al., 2003). The median values of bootstrap estimates along with their corresponding 95% confidence intervals were compared to those estimated from the original dataset in order to assess the stability of the model. Furthermore, a prediction-corrected visual predictive check (pc-VPC) was employed to evaluate the predictive performance of the final model (Bergstrand et al., 2011). A comparison was made based on 1,000 simulations to visually assess whether the median, 5^th^ and 95^th^ percentiles of prediction-corrected observed data fell within the 90% confidence interval of corresponding percentiles of simulated data.

### Simulations for dose selection

A total of 1,000 subjects were randomly sampled with replacement from the study cohort. Simulations based on the final PK-PD model were performed to illustrate the impact of different dose regimens on predicted CD19^+^ lymphocyte counts. Six dose scenarios, including a single infusion of 100 mg/m^2^, a single infusion of 375 mg/m^2^, two weekly infusions of 375 mg/m^2^, four weekly infusions of 375 mg/m^2^, a single infusion of 750 mg/m^2^ and two infusions of 750 mg/m^2^ given 2 weeks apart, were simulated to compare the time course of CD19^+^ lymphocyte suppression in this pediatric cohort with FRNS/SDNS. B-cell recovery was defined as peripheral CD19^+^ cell count exceeding 10 × 10^6^/L.

## Results

### Patients and data collection

**FIGURE 2:**
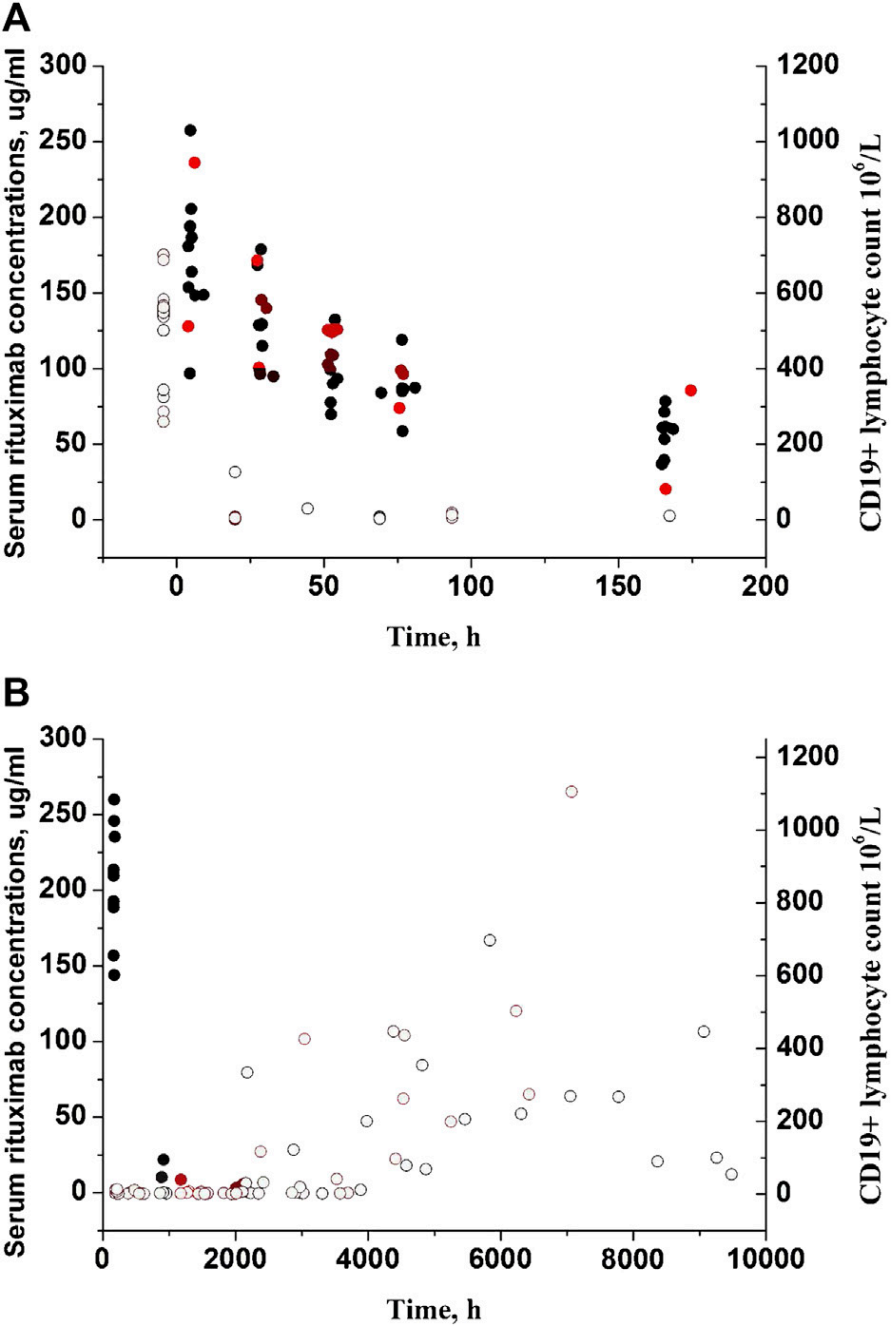
Individual serum rituximab concentrations and CD19^+^ counts with time profiles. **(A)** Plots after the first dose. **(B)** Plots after the last dose. Closed circles represent rituximab concentrations previously reported in our study (Chen et al., 2021), while open circles represent CD19^+^ counts. Black circles represent patients receiving two infusions of rituximab, and red circles represent patients receiving only one infusion.

Fourteen pediatric patients diagnosed with FRNS/SDNS were enrolled in this study. Rituximab was administered intravenously at a dose of 375 mg/m^2^ (a maximum of 500 mg) for a duration of 2 weeks. Among the 14 patients, eleven received two infusions of rituximab, while three children received only one infusion due to proteinuria recurrence. Their clinical and demographic characteristics have been published in our previous paper (Chen et al., 2021). The changes of CD19^+^ lymphocytes and levels of anti-rituximab antibody prior to medication or during follow-up are summarized in Table 1. A total of 102 measurements of CD19^+^ lymphocyte counts were available for PK-PD analysis. No detectable levels of anti-rituximab antibodies were observed during the PK follow-up period. The temporal dynamics of CD19^+^ counts are illustrated in Figure 2. In order to comprehensively examine the concentration-response relationship, we integrated individual serum rituximab concentrations from our previous study into Figure 2 (Chen et al., 2021).

**TABLE 1:** Changes of CD19^+^ lymphocytes and levels of anti-rituximab antibody in pediatric patients receiving rituximab treatment.

Variable	Value/median [range]
No. of patients/CD19+ counts	14/102
Baseline CD19^+^ lymphocytes (10^6^/L)	548.0 [258.4–701.6]
CD19^+^ lymphocytes post-administration (10^6^/L)	10.97 [0–1,104.6]
CD19^+^ lymphocytes before B-cell recovery^a^ post-administration (10^6^/L)	3.4 [0–125.9]
CD19^+^ lymphocytes after B-cell recovery^a^ post-administration (10^6^/L)	200.6 [10.9–1,104.6]
Time to CD19^+^ lymphocytes nadir (days)	37.5 [1–81]
Time to B-cell recovery^a^ ^,^ ^b^ (days)	154.5 [8–395]
Anti-rituximab antibody sampling time (days)	7.2 [3.2–88]
Anti-rituximab antibody (Positive/Negative)	0/14

^a^ B-cell recovery was defined as peripheral CD19^+^ lymphocytes exceeding 10 × 106/L.

^b^ Two pediatric patients with no observed B-cell recovery during the follow-up were excluded from the estimates.

### Population PK-PD modelling

The serum concentrations were best described by a 2-compartment PK model (Chen et al., 2021). The PD process was best described by a turnover model incorporating rituximab promotion on CD19^+^ lymphocytes elimination (K_out_). Residual variability in both serum concentration and CD19^+^ lymphocytes was characterized using a proportional error model. None of the covariates investigated exhibited a statistically significant impact on the depletion of CD19^+^ lymphocytes. A summary of all PK-PD model parameters can be found in Table 2.

**TABLE 2:** Parameter estimates of rituximab final PK-PD model and bootstrap validation.

Parameters	Estimated value (RSE%)	IIV(RSE%)	Bootstrap of estimates median (95%CI)	Bootstrap of IIV median (95%CI)	Shrinkage
PK model					
CL (mL/h)	8.69^a^ (fixed)	37.0% (101.5)	NA	33.3% (12.7%–97.3%)	4.8%
V_1_ (L)	1.86^a^ (fixed)	25.4% (131.4)	NA	23.4% (9.3%–32.1%)	7.8%
Q (mL/h)	7.5^a^ (fixed)	NE	NA	NA	NA
V_2_ (L)	1.9^a^ (fixed)	NE	NA	NA	NA
*θ*_BSA∼CL_	1.26^a^ (fixed)	NA	NA	NA	NA
σPK	0.18 (30.2)	NA	0.19 (0.14–0.29)	NA	12.8%
PD model					
E_MAX_	99.6 (84.7)	NE	103 (78.1–153)	NA	NA
EC_50_ (µg/mL)	5.87 (50.3)	NE	5.38 (1.56–31.6)	NA	NA
BSLN (10^6^/L)	395 (124.8)	65.8% (155.7)	405 (331–532)	NA	1.8%
K_OUT_ (/day)	0.051 (136.3)	65.4% (259.3)	0.052 (0.026–20.3)	NA	42.2%
σPD	0.84 (49.4)	NA	0.81 (0.69–0.94)	NA	4.6%

^a^ Parameters derived from our previous publication (Chen et al., 2021).

CL, central clearance; V_1_, central volume of distribution; Q, inter-compartment clearance; V_2_, peripheral volume of distribution; E_MAX_, maximum cell killing effect of rituximab; EC_50_, rituximab concentration at 50% of maximum effect; BSLN, CD19^+^ lymphocyte counts at baseline; K_OUT_, death rate constant of CD19^+^ lymphocytes; RSE%: relative standard errors; IIV: Inter-individual variability; CI: confidence interval; NE: not estimate; NA: not applicable.

### Model diagnosis and evaluation

**FIGURE 3:**
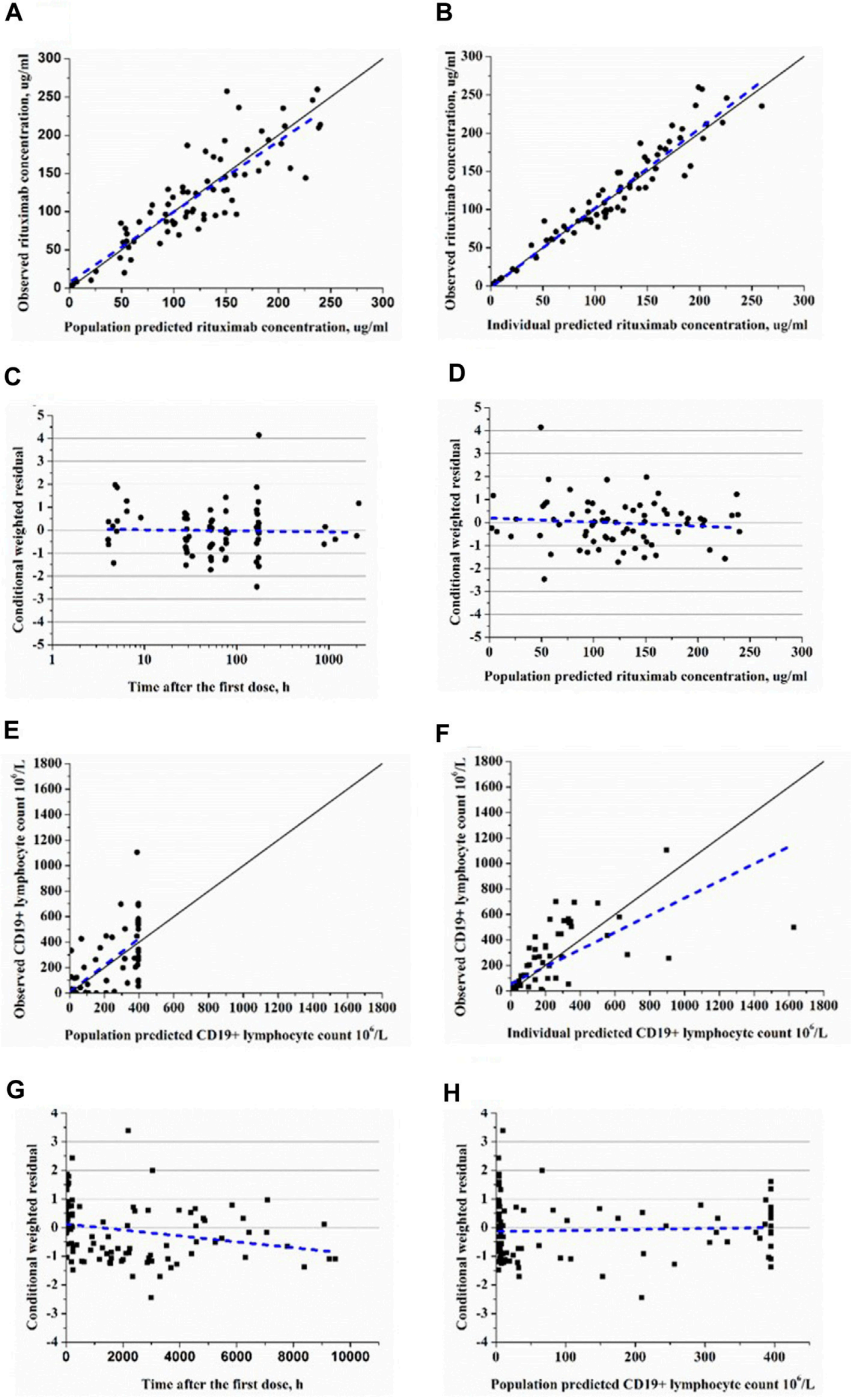
Goodness-of-fit plots of rituxiamb final PK-PD model. **(A)** The observed *versus* population-predicted concentration. **(B)** The observed *versus* individual-predicted concentration. **(C)** Conditional weighted residual *versus* time. **(D)** Conditional weighted residual *versus* the predicted concentration. **(E)** The observed *versus* population-predicted CD19^+^ lymphocyte count. **(F)** The observed *versus* individual-predicted CD19^+^ lymphocyte count. **(G)** Conditional weighted residual *versus* time. **(H)** Conditional weighted residual *versus* the predicted CD19^+^ lymphocyte count.

**FIGURE 4:**
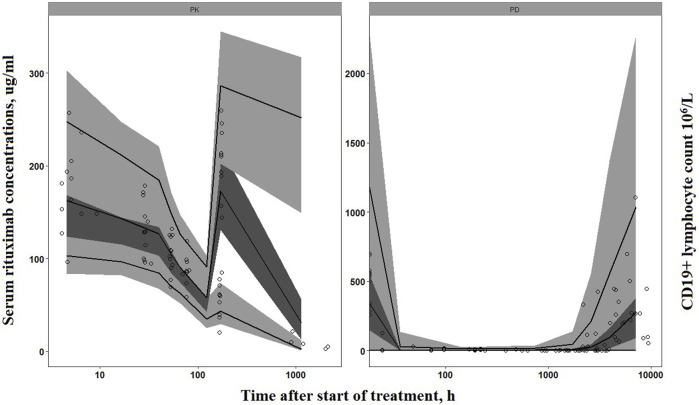
Prediction-corrected visual predictive check of the final PK-PD model. Circles represent the observed concentrations (rituximab concentrations have been reported in our previous study (Chen et al., 2021)). Solid lines represent the observed median, 10th and 90th percentiles of observed profiles. The shaded areas correspond to the simulation-based 95% confidence intervals.

The goodness-of-fit plots demonstrated that the final PK-PD model adequately predicted the observed rituximab concentration and CD19^+^ lymphocyte count (Figure 3). Plots of population- and individual-predicted values *versus* observed values exhibited no significant bias. The conditional weighted residuals displayed a symmetrical distribution around zero, with the majority of points falling within an acceptable range of ±2. The results of 1,000 bootstrap replicates for rituximab are summarized in Table 2. The median parameter estimates obtained from bootstrap validation were close to the values derived from the final model, while the 95% confidence intervals (CI) of bootstrap parameters encompassed all values from the final model, indicating robust stability and precision of the final PK-PD model. Figure 4 presents the pc-VPC plot for the final model. The agreement between prediction-corrected observed median, 5th and 95th percentiles with simulated counterparts further confirms good predictive performance of the final PK-PD model.

### Simulations for dose selection

**FIGURE 5:**
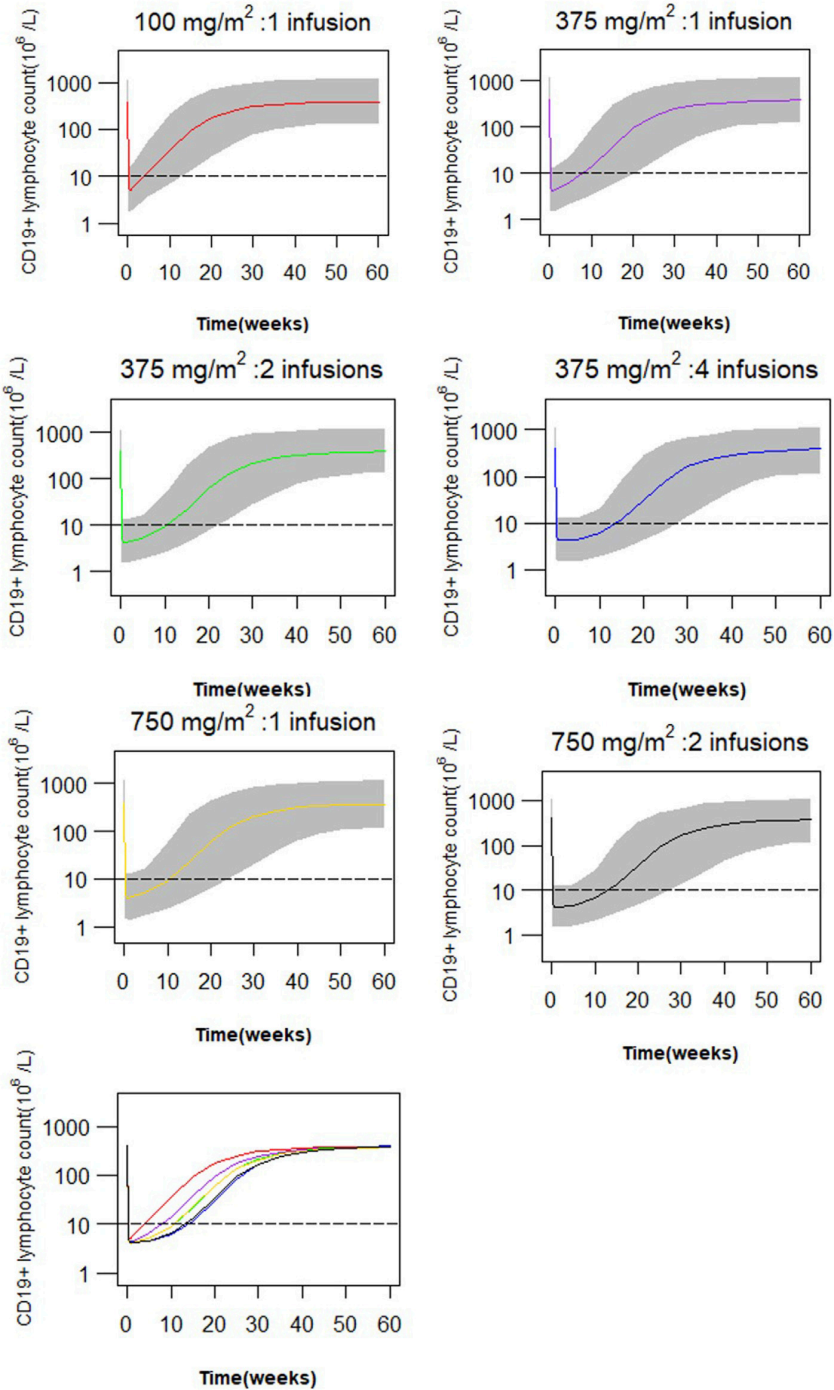
Simulated profiles of suppression of CD19^+^ lymphocytes after variable dosing regimen of rituximab (red: a single infusion of 100 mg/m^2^, purple: a single infusion of 375 mg/m^2^, green: 2 infusions of 375 mg/m^2^ given 2 weeks apart, blue: four weekly infusions of 375 mg/m^2^, gold: a single infusion of 750 mg/m^2^, black: 2 infusions of 750 mg/m^2^, grey band: 90% prediction interval).

Simulations of the time course of CD19^+^ lymphocytes for the six dosing regimens are presented in Figure 5. A single infusion of 100 mg/m^2^ resulted in near-complete depletion of CD19^+^ lymphocytes following rituximab treatment, and the same maximum suppression was achieved with 4 weekly 375 mg/m^2^ infusions or 2 weekly 750 mg/m^2^ infusions. While there is a variation in the extent of CD19^+^ lymphocyte suppression among different dosing regimens within 4 weeks, all regimens effectively maintain CD19^+^ cell counts below 10 × 10^6^/L along a similar gradient. Comparable responses with respect to peripheral blood CD19^+^ lymphocytes can be attained through two infusions of 375 mg/m^2^ or a single infusion of 750 mg/m^2^, and an equivalent effect can be achieved by administering four infusions of 375 mg/m^2^ as compared to two infusions of 750 mg/m^2^. However, lower doses lead to more rapid recovery of CD19^+^ lymphocytes. The duration of CD19^+^ lymphocyte suppression (<10 × 10^6^/L) following a single infusion of 100 and 375 mg/m^2^ accounted for 37% and 76%, respectively, compared to the duration of suppression after two weekly infusions of 375 mg/m^2^. Simulations of suppression time to reach lymphocyte counts of 10 × 10^6^/L, 100 × 10^6^/L or 200 × 10^6^/L are given in Table 3.

**TABLE 3:** Simulated suppression time to recovery to 10 × 10^6^/L, 100 × 10^6^/L or 200 × 10^6^/L in weeks for pediatric patients with frequent-relapsing or steroid-dependent nephrotic syndrome.

Dosing	Duration (90% PI) below 10 × 10^6^/L (weeks)	Duration (90% PI) below 100 × 10^6^/L (weeks)	Duration (90% PI) below 200 × 10^6^/L (weeks)
100 mg/m^2^, 1 infusion	4.0 (0–13.0)	15.7 (7.1–36.1)	22.0 (9.7-∞)
375 mg/m^2^, 1 infusion	8.2 (0–20.6)	20.7 (9.8–43.1)	27.1 (12.9-∞)
375 mg/m^2^, 2 infusions	10.8 (0–22.2)	23.2 (12.7–44.3)	28.9 (15.0-∞)
375 mg/m^2^, 4 infusions	14.1 (0–27.4)	26.4 (15.6–49.3)	32.1 (18.4-∞)
750 mg/m^2^, 1 infusion	10.2 (0–23.8)	23.2 (12.2–46.2)	29.4 (14.7-∞)
750 mg/m^2^, 2 infusions	12.9 (0–27.4)	25.9 (14.3–52.5)	32.1 (17.4-∞)

PI, prediction interval.

## Discussion

The off-label use of rituximab has demonstrated promising results with variable response rates in children with FRNS/SDNS. Direct comparision between different regimens across studies is challenging due to variations in dosing, frequency and timing of administration. The appropriate dosing schedule for pediatric patients with FRNS/SDNS remains unclear, necessitating further modifications to achieve sustained remission and reduce relapse rates. This study presents the first population PK-PD model for rituxiamb and conducted simulations with different dosing scenarios in this population. CD19^+^ lymphocytes were used as the PD marker to evaluate the PK-PD relationship of rituximab. Consistent with previous studies, we observed rapid and near-complete depletion of CD19^+^ lymphocytes following rituximab treatment despite a wide range of initial CD19^+^ lymphocyte counts (258.4–701.6 × 10^6^/L). Our proposed PK-PD model incorporating a turnover mechanism effectively describes changes in CD19^+^ lymphocytes after rituximab treatment. The much lower estimated E_max_ value of 99.6 × 10^6^/L indicates the potential for significant lymphocyte depletion compared to the median baseline CD19^+^ lymphocyte count of 548.0 × 10^6^/L. The estimated EC_50_ is 5.87 μg/mL, which is consistent with the range of 2–10 μg/mL *in vitro* studies (Vugmeyster et al., 2003). Furthermore, we investigted the impact of covariates on rituximab PD. Morbach et al. reported age-related decreases in total CD19^+^ B cells within lymphocytes (Morbach et al., 2010). However, our current study did not identify the covariate effect of age on rituximab PD probably due to the limited sample size of pediatric patients. Thus, larger cohorts covering a wider range of ages may be needed to observe and quantify age-dependent cell growth. Similarly, we conducted covariate analyses using proteinuria (positive/negative) or proteinuria-to-creatinine ratio to examine the influence of proteinuria on PD. However, neither proteinuria nor proteinuria-to-creatinine ratio showed a significant effect when included as covariates. This finding may be attributed to the characteristics of our study population. Prior to receiving maintenance therapy with rituximab, induction treatment was administered either with corticosteroids alone or in combination with other immunosuppressants such as cyclosporine, mycophenolate mofetil, and tacrolimus. All pediatric patients enrolled in this study had negative proteinuria at baseline, and only three children experienced mild proteinuria during follow-up. Simulations were conducted to compare the effects of different dose regimens of rituximab on the time course of CD19^+^ lymphocytes. Upon finding a low EC_50_ value, we hypothesized that a similar effect could be achieved with a lower dose. A multicenter retrospective observational cohort study conducted by Maxted et al. indicated that the median time to reconstitution of B cells was not significantly different in children with FRNS who received seven different dosing regimens ranging from 375 to 750 mg/m^2^, administered as 1-4 doses within 12 months, and a single low-dose regimen of 375 mg/m^2^ did not affect the time to B cell reconstitution compared to a conventional higher dose (Maxted et al., 2019). In contrast, Hogan et al. in their retrospective study comparing three different dosing strategies in SDNS patients, demonstrated that the time to B cell reconstitution increased when receiving one injection of 100 mg/m^2^, one injection of 375 mg/m^2^ and two injections of 375 mg/m^2^. The use of a single dose of 100 mg/m^2^ was associated with shorter duration of B cell depletion, and thus appeared to increase the risk of earlier relapse (Hogan et al., 2019). It should be noted that both studies had limited sample sizes in their low-dose and high-dose groups. In our present study, we found that treating children with FRNS/SDNS using a single dose of 100 mg/m^2^, 375 mg/m^2^ or 750 mg/m^2^ resulted in similar treatment effects as observed by CD19^+^ lymphocyte suppression within 4 weeks at mostly identical levels (less than 10 × 10^6^/L). However, the time for B cell recovery decreased with lower doses of rituximab in children with FRNS/SDNS and lower doses were associated with shorter durations for CD19^+^ lymphocyte depletion consistent with the results reported by Hogan et al. (2019). The difference lies in their findings showing shorter times for B cell reconstitution when using one dose of 100 mg/m^2^, one dose of 375 mg/m^2^ or two doses of 375 mg/m^2^ compared to our cohorts (2.5 months vs. 5.5 months, 5.0 months vs. 6.8 months and 6.6 months vs. 7.2 months, defined as CD19^+^ B cells greater than 200× 10^6^/L). Higher doses or more frequent regimens may enhance the likelihood of response in pediatric patients with FRNS/SDNS. Rituximab has demonstrated favorable tolerability in most published studies involving its use for FRNS/SDNS treatment. Administering a lower dose of rituximab would yield reduced treatment costs. However, B cell reconstitution resulting from lower doses necessitates repeated rituximab injections, which may potentially induce anti-rituximab antibodies as previously reported in children with NS (Ahn et al., 2014; Fujinaga et al., 2020). Therefore, further investigation is required to determine the optimal dosing regimen.

Several limitations of this study should be taken into consideration. Firstly, the sample size was relatively small, which may have limited the ability to detect certain covariates that could be better quantified in large-scale studies. However, population PK-PD analysis can be conduted using sparse and unbalanced data by leveraging information from different individuals. In our study, the estimated parameters were consistent with values reported in existing literature, indicating the reliability of our data and validity of our model. The inter-individual variability of PK and PD parameters also remained within a reasonable range. Model evaluations further supported the good stability and predictive performance of the final PK-PD model. Secondly, the correlations between CD19^+^ levels and treatment responses are still needed to validate through larger studies. While a few patients may experience NS relapses despite complete depletion of CD19^+^ B cells (Kamei et al., 2009; Sato et al., 2018), there are also cases where long-term clinical remission is maintained even after total B-cell recovery (Sel et al., 2012). Nevertheless, CD19^+^ B-cell depletion remains one of the most commonly used biomarkers for monitoring rituximab treatment in NS, as numerous studies have shown that most NS relapses occur after B cell recovery. At our center, the monitoring of B cell depletion and reconstitution has proven encouraging and helpful in guiding decisions regarding additional rituximab doses. Finally, we did not assess anti-rituximab antibody levels beyond 90 days. Typically, in the majority of patients undergoing rituximab treatment, the development of anti-rituximab antibodies is observed approximately 3 months after administration when rituximab concentrations have declined (Oomen et al., 2022; Saffari and Jafarzadeh, 2022). Therefore, Long-term monitoring of anti-rituximab antibodies is warranted.

## Conclusion

In summary, we present a population PK-PD model of rituximab for the first time and provide a potential pathway for future model guided dosing strategy for therapy in pediatric patients with FRNS/SDNS. Our modeling and simulation analysis suggest that administering two infusions at a dose of 375 mg/m^2^ or a single infusion at a dose of 750 mg/m^2^ would result in similar effects on CD19^+^ B cells (<10 × 10^6^/L). Similarly, four infusions at a dose of 375 mg/m^2^ or two infusions at a dose of 750 mg/m^2^ would yield comparable 10-week suppression of CD19^+^ lymphocytes. Further clinical studies are warranted to evaluate the efficacy and safety of different dosing schemes.

## Data Availability

The raw data supporting the conclusion of this article will be made available by the authors, without undue reservation.
